# Data-driven approaches for predicting mechanical properties and determining processing parameters of selective laser sintered nylon-12 components

**DOI:** 10.1007/s44245-025-00094-7

**Published:** 2025-03-22

**Authors:** Ruixuan Tu, Candice Majewski, Inna Gitman

**Affiliations:** 1 Division of Surgery and Interventional Science, University College London, London, UK; 2 Department of Mechanical Engineering, University of Sheffield, Sheffield, UK; 3 Department of Mechanics of Solids, Surfaces & Systems, University of Twente, Enschede, The Netherlands

**Keywords:** Selective laser sintering, Fuzzy inference system, Neural networks, Adaptive neural fuzzy inference system, Polymer additive manufacturing

## Abstract

In order to allow engineers to make decisions regarding laser settings in selective laser sintering and predict the mechanical properties of materials, conventional material models could provide accurate solutions and recommendations, however, they are potentially expensive and time-consuming. Thus, a number of computational data-driven methodologies have been introduced in this article, as alternatives, to formulate cross-correlations between the processing parameters and mechanical properties of selective laser sintered (SLS) nylon-12 components. Proposed in this article *direct*—from laser settings to material properties, and *inverse*—from desired material properties to laser settings, two estimation frameworks have provided accurate estimation results. The accuracy of three proposed data-driven methodologies: fuzzy inference system (FIS), artificial neural networks (ANN) and adaptive neural fuzzy inference system (ANFIS), have been compared and thoroughly analysed, with FIS being the most accurate solution.

**Supplementary Information:**

The online version contains supplementary material available at 10.1007/s44245-025-00094-7.

## Introduction

Selective laser sintering (SLS) is one of the powder-based additive manufacturing (AM) techniques which is used to manufacture lightweight, rigid components including rapid prototypes and functional production parts. Developed in early 2000s, SLS employs a high-power laser to selectively sinter powder particles until they have melted enough for the particles to join together, creating the predetermined shape [1, 2]. Once the shape is formed in the current layer, the build chamber lowers so that the additional layer of powders can be distributed on top of the finished one for further sintering. The process is repeated layer by layer until the build is complete. Different from the conventional subtractive manufacturing techniques, SLS allows components to be manufactured additively and selectively, leading to less material waste, saving on cost for industry and more flexible design [2].

Despite many advantages of SLS, the additively manufactured parts can still have various unique defects which are different from those in their cast and wrought counterparts. These defects include porosity due to unmelted powders and gas entrapment, anisotropy of microstructure and tensile strength, and distortion caused by large residual stress due to rapid cooling process [3]. Aforementioned defects could potentially lead to undesired and possibly unpredicted mechanical behaviour. Hence, it is of great importance to understand the complex relationship between the printing process and the mechanical properties of SLS parts. As shown in previous studies [4–7], typical laser-related process parameters include laser power (LP), laser speed (LS) and scan spacing (SS), which all have a significant influence on the quality of printed parts.

In the past years, multiple studies have been conducted to analyse the individual effect of aforementioned parameters. Hou et al. [8, 9] have noted that the increasing LP, i.e. sufficient energy during powder sintering, can lead to a larger tensile strength. Gharate et al. [10] have reported that a decreased sinterability can be achieved at greater LP and layer thickness. Besides a tensile strength, Magri et al. [11] have demonstrated that both Young’s modulus (YM) and elongation at break (EaB) increase with a higher LP. Ullah and Rehman [12] reported that lower LS allow material powders to absorb more heat and melt properly, but molten droplets generated at lower LS may cause unexpected defects between layers. Singh et al. [13] concluded in their research that the lower SS can result in significant increase of density and hardness of the printed parts.

Analysing the above literature, it can be seen that the effect of individual process parameters on mechanical properties can already be cumbersome to predict; taking into account multiple process characteristics simultaneously and cross-correlation of these process characteristics could become even more convoluted, especially using available to-date physics-based constitutive equations.

As an alternative to physics-based equations, data-driven approaches have recently raised increasing interests in the research community. These approaches gain knowledge directly from data and “learn” the patterns, which can be used to interpret the vagueness in data and predict further behaviour. Typical data-driven methods include artificial neural networks (ANN) [14, 15], fuzzy inference system (FIS) [16–18], adaptive neural fuzzy inference system (ANFIS) [19, 20] and other techniques.

Several attempts have been made to-date in applying data-driven methods to analyse SLS. For example, Dingal et al. [21] applied Taguchi method, together with analysis of variance (ANOVA) to investigate how density, porosity and hardness of SLS parts are affected by process parameters such as LP, LS, layer thickness. Negi et al. [22] applied and compared both response surface methodology (RSM) and ANN approach to observe the influence of bed temperature, LS and SS on shrinkage. Sohrabpoor et al. [23] employed ANFIS to generate a mapping relationship between process parameters, such as bed temperature, LP, LS and SS, and response outputs, including elongation and ultimate tensile strength (UTS). FIS approach has also shown its reliability in previous studies to formulate the relationship between fused deposition modelling (FDM) process parameters and mechanical properties [16, 17, 24].

Interesting to note, that all aforementioned prior studies have focused on individual methodologies to model relationship between process parameters and mechanical properties. In this work we see the novelty in not only demonstration of the ability of the data-driven methodologies to analyse SLS, but also in providing a comparative evaluation of these methods for a better understanding of which approach yields the most accurate results.

What is possibly even more appealing beyond the analysis of accuracy, is the investigation of optimal combinations of process parameters leading to desired mechanical properties—so-called *inverse* problem. In this paper, both frameworks will be introduced: (i) *direct* framework, where process parameters are taken as inputs to estimate the corresponding mechanical response; and (ii) *inverse* framework, where mechanical properties, e.g. potential real-life industrial requirements, are used as inputs to estimate the optimal combination of SLS process parameters.

Thus, this research offers a detailed comparative analysis of multiple data-driven approaches, applied to analyse SLS process, offering insights into their predictive capabilities with the assist of two novel estimation frameworks. The study presented in this paper, evaluates the performance of three specific data-driven approaches: FIS, ANN and ANFIS, providing a foundation for more effective decision-making in the application of data-driven solutions to SLS processes.

In the first part of Sect. 2 a brief introduction will be given to the adopted data-driven approaches (FIS, ANN and ANFIS). Later in this section the experimental and testing setup will be presented. Section 3 will discuss estimation results of all three approaches in both direct and inverse frameworks. A comparative study of all three methodologies will be discussed in Sect. 3 as well.

## Methodologies and experimental setup

Although not novel in itself, for the completeness of the paper, this section starts with a brief introduction to the data-driven methodologies adopted in the present investigation, namely FIS, ANN and ANFIS.

### Artificial neural networks (ANN)

The first methodology that will be discussed is Artificial Neural Networks (ANN)—this methodology is used more frequently than other data-driven approaches and is often used synonymously with data-driven models. However, as will be evident from the discussion below, ANN is a specific example of data-driven models. ANN is a mathematical model which simulates the signal-transmitting patterns between biological neurons in human brains [25]. Typically, an ANN consists of several interconnected artificial neurons, each containing information from the previous neurons. The information from preceding neurons has an associated weighting value and the current neuron’s output is the weighted sum of all previous inputs. Initially, these weights may not provide accurate estimations; hence the key idea of ANN is to update weight values through iterative calculations aimed at minimising the error between the estimated value and the target value until an acceptable accuracy is achieved. Such iterative calculations are normally referred to as the learning process of ANN and the final trained network with updated weights can be utilised for estimation purposes.

ANNs excel at modelling complex non-linear relationships in data with intricate dependencies, which in some cases cannot be captured by models such as Support Vector Regression (SVR) or traditional Gaussian Process Regression (GPR) [26, 27]. Compared with other data-driven methods, ANN benefits from its flexible architectures which can be tailored for various types of data, e.g. images and sequential data [28]. Additionally, it demonstrates scalability with large datasets when employing deep learning architectures [29]. Thus, ANN is applicable in this research to facilitate the prediction of various mechanical properties based on varying manufacturing parameters (direct) and to identify the combination of manufacturing parameters with mechanical requirements (inverse).

However, as ANN is one of the “black-box” models, the recursive learning process is difficult to interpret compared with models like Fuzzy Inference Systems (FIS) to be introduced later, which offer clear decision-making rules [30]. Furthermore, the requirement of significant computational resources and training time [31], along with the need for large datasets to perform effectively [32], poses limitations, especially when compared with fast models like FIS.

### Fuzzy inference system (FIS)

As a compelling alternative to ANN, Fuzzy Inference System is also employed in this research as a prominent data-driven approach. FIS is grounded in the theory of fuzzy sets and fuzzy logic, first proposed by Zadeh [33, 34]. Similar to the human reasoning process where a description can be partly true or false, FIS allows for degrees of membership in variables, facilitating a more nuanced representation of uncertainty and vagueness. This makes FIS particularly robust in environments where data is imprecise, subjective or noisy [35].

The membership degree in fuzzy logic ranges from 0 to 1, in contrast to traditional binary sets, which only take on values of 0 or 1. In a fuzzy system, historical data is translated into fuzzy rules, enabling the estimation of outputs based on given new inputs. These rules effectively capture expert knowledge and linguistic descriptions, making FIS particularly valuable in applications where insights about uncertainties are critical. One of the benefits of employing FIS is its transparent and interpretable process.

One prominent model within FIS is the Sugeno fuzzy system, which uses mathematical functions (either linear or constant) as output rather than fuzzy sets, as seen in a Mamdani Fuzzy System [16]. This distinction enhances the Sugeno model’s compatibility with other machine learning techniques, such as ANN, leading to the development of Adaptive Neuro-Fuzzy Inference System (ANFIS). Additionally, the structure of Sugeno model makes it more computationally efficient, allowing for faster processing and easier integration with numerical optimisation methods [36]. Hence, FIS approach is applicable in this research considering its computational efficiency and imprecision allowance in complex manufacturing processes.

However, unlike ANN model, FIS may struggle with scalability when dealing with high-dimensional datasets, which may lead to increased computational complexity [37]. Furthermore, compared to ANN, FIS may be less flexible in adapting to complex, non-linear relationships, as it relies heavily on predefined rules that may not capture all data patterns [38]. Therefore, to address some of these limitations, ANFIS will be introduced in the following section.

### Adaptive neural fuzzy inference system

FIS approach is able to handle inaccurate or indeterministic inputs by including engineering uncertainties in the form of membership degrees. However, there are no effective standard solutions to transform human knowledge into fuzzy rules and data. Hence, with the recursive learning process of ANN, parameters in FIS can be determined more accurately. With this idea, Jang [36] first proposed an approach known as Adaptive Neural Fuzzy Inference System (ANFIS), which integrates the uncertainty reasoning process of FIS and iterative learning and connection pattern of ANN. ANFIS has a similar “layers of neuron” structure to ANN whereas the information in each neuron is linked with every fuzzy rule, i.e. membership values. This hybrid approach enables ANFIS to learn from input–output data and adapt its fuzzy rules, accordingly, making it particularly effective for complex, non-linear problems. Hence, with the ability to capture complex non-linear relationships between varying manufacturing parameters, along with the interpretability of learning process, ANFIS is selected in this study as another data-driven approach.

Similar to ANN, training an ANFIS model can be computationally intensive, especially when dealing with large datasets using iterative calculations [39]. Furthermore, the performance of ANFIS is dependent on the quality of the initial fuzzy rules and membership functions (MF) [40].

All three aforementioned approaches will be tested in this paper and the comparative analysis of these methodologies will conclude if any of these approaches could be chosen as the preferred one in analysing and predicting mechanical properties, along with the combination of manufacturing parameters of SLS parts.

### Experimental setup and sample tests

**Fig. 1:**
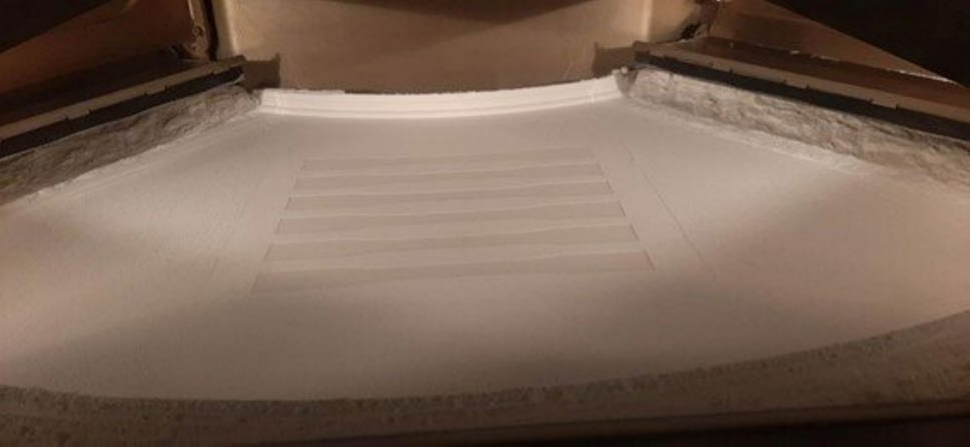
Building layout of specimens being printed

SLS samples were produced in PA2200 (nylon-12) powder, at a ratio of 50:50 virgin to ‘used’ powder, on an EOS Formiga P100 Laser Sintering system, see Fig. 1. As previous trials within our research team suggested, all samples were printed with a layer thickness of 0.1mm and a part bed temperature of $170^\circ{\rm C}$, the temperature to which powder is preheated. To investigate the effects of a range of processing parameters, parts were manufactured at varying combinations (high, medium and low) of LP, LS, and SS, see Table 1. The range of printing parameters selected was based upon previous (unpublished) research within our team, which confirmed that these parameters would provide a useful variety of mechanical properties, while ensuring that all parameter sets were manufacturable and would produce robust parts.

**Table 1:** Processing parameters in the printing process

Laser power (LP) [W]	High = 24	Medium = 21	Low = 18	Higher laser power = higher energy input
Laser speed (LS) [mm/s]	High = 3000	Medium = 2500	Low = 2000	Higher laser speed = lower energy input
Scan spacing (SS) [mm]	High = 0.3	Medium = 0.25	Low = 0.2	Higher scan spacing = lower energy input

ASTM D638 type I tensile specimens were produced in 15 layers of 6 samples and left to cool to room temperature before being removed from the build chamber. Finally, parts were de-powdered using compressed air.

Tensile testing was carried out on a Tinius Olsen 5K with Laser Extensometer, in accordance with ASTM D638, at a rate of 5 mm/min (see results of the tension test in Table 2). Note that each combination of process parameters was repeated three times, values presented in Table 2 are the averages of these three repeats. The vertical position (VP) of samples also had three different levels—bottom (within the first five layers of parts), middle (within layers 6–10) and top (layers 11–15), quantified as “1” (bottom), “2” (middle) and “3” (top)” in Table 2. Summarising, each of 4 parameters (LP, LS, SS, VP) had three different levels, resulting in 81 tested samples (as also follows from the design of experiment standard practice); however, due to an unexpected fault with the tensile test system, some samples have been excluded from the analysis, leaving 74 data sets (each representing a unique combination of processing parameters), see Table 2. To maximise the accuracy of predictions, and to cover a wide range of parameter combinations, capturing essential features of the design space, it has been decided to consider the full 74 data sets in the study. Note, however that the methodology to estimate the sufficient size of the data set can be found, for example in [16, 41].

**Table 2:** Experimental results of printed parts

Sample	Laser power (W)	Laser speed (mm/s)	Scan spacing (mm)	Vertical position	Ultimate tensile strength (MPa)	Young's modulus (MPa)	Elongation at break (%)
1	18	2000	0.2	1	48.9	1860	25.2
2	18	2000	0.2	2	49.3	1680	31.1
3	18	2000	0.2	3	47.4	1930	31.9
4	18	2000	0.25	1	49.2	1800	25.3
5	18	2000	0.25	2	47.7	1890	23.8
6	18	2000	0.25	3	46.9	1740	27.2
7	18	2000	0.3	1	47.9	1870	22.4
8	18	2000	0.3	2	46.7	1840	19
9	18	2000	0.3	3	46	1820	22.9
10	18	2500	0.2	1	49.8	1700	31.6
11	18	2500	0.2	2	48.2	1900	25.3
12	18	2500	0.2	3	47.8	1660	29.7
13	18	2500	0.25	1	46.9	1720	18.3
14	18	2500	0.25	2	46.3	1710	22
15	18	2500	0.25	3	46.3	1640	18.2
16	18	2500	0.3	1	43.4	1620	13.1
17	18	2500	0.3	2	43	1680	13.1
18	18	2500	0.3	3	42.4	1690	13.9
19	18	3000	0.2	1	48	1760	21
20	18	3000	0.2	2	47.7	1900	20.8
21	18	3000	0.2	3	46.4	1720	22.9
22	18	3000	0.25	1	43.4	1710	11.3
23	18	3000	0.25	2	41.6	1550	12.1
24	18	3000	0.25	3	40.9	1670	10.9
25	18	3000	0.3	1	31.4	1240	8.1
26	18	3000	0.3	2	31.6	1310	8.5
27	18	3000	0.3	3	32.4	1320	8.4
28	21	2000	0.2	1	45.7	1700	26.9
29	21	2000	0.2	2	46.5	1700	24.8
30	21	2000	0.2	3	45	1720	29.2
31	21	2000	0.25	1	48.8	1940	26.9
32	21	2000	0.25	2	48.2	1690	24.2
33	21	2000	0.25	3	47.5	1920	23.9
34	21	2000	0.3	1	48	1690	23.7
35	21	2000	0.3	2	48	1730	27
36	21	2000	0.3	3	47.7	1710	26.4
37	21	2500	0.2	1	48.6	1940	24.6
38	21	2500	0.2	2	48.8	1860	34.4
39	21	2500	0.2	3	47.9	1850	27.3
40	21	2500	0.25	1	48	1740	25.6
41	21	2500	0.25	2	47.5	1740	21.4
42	21	2500	0.25	3	47	1840	25.7
43	21	2500	0.3	1	46.6	1660	19.3
44	21	2500	0.3	2	46.5	1690	16.4
45	21	2500	0.3	3	46	1780	21.3
46	21	3000	0.2	1	47.8	1830	27
47	21	3000	0.2	2	46.9	1820	26
48	21	3000	0.2	3	46.9	1710	25.8
49	21	3000	0.25	1	47.8	1760	19.7
50	21	3000	0.25	2	45	1650	17.7
51	21	3000	0.25	3	45.8	1640	21.7
52	21	3000	0.3	1	40.1	1580	11.8
53	21	3000	0.3	2	40.3	1530	10.6
54	21	3000	0.3	3	35.3	1310	9.3
55	24	2000	0.2	1	45.4	1580	28.3
56	24	2000	0.2	2	47.1	1720	25.6
57	24	2000	0.2	3	44.1	1670	29.3
58	24	2000	0.25	1	47.3	1740	27.6
59	24	2000	0.25	2	48.4	1820	25.9
60	24	2000	0.25	3	48	1790	26.8
61	24	2000	0.3	2	47.9	1740	24.2
62	24	2000	0.3	3	47.1	1770	29.3
63	24	2500	0.2	2	48	1780	28.8
64	24	2500	0.2	3	46.6	1710	25.7
65	24	2500	0.25	2	48.7	1820	26.5
66	24	2500	0.25	3	47.2	1640	27.8
67	24	2500	0.3	2	47.2	1630	25
68	24	2500	0.3	3	45.8	1720	23.7
69	24	3000	0.2	2	48	1630	26.9
70	24	3000	0.2	3	46.9	1660	31
71	24	3000	0.25	2	47.2	1670	19.8
72	24	3000	0.25	3	46.7	1710	19.9
73	24	3000	0.3	2	45.5	1790	15.1
74	24	3000	0.3	3	44.4	1650	17.7

## Results and discussions

As mentioned in the Introduction, the main focus of this paper is to evaluate the capability of data-driven techniques to formulate relationships between mechanical properties of printed parts and SLS process parameters. Mathematically speaking, these formulations involve predicting output characteristics based on input data for both the direct and the inverse frameworks. In the direct framework, LP, LS, SS, and VP are the input parameters, while UTS, YM, and EaB are the output parameters. Conversely, in the inverse framework, UTS, YM and EaB, acting as input parameters, provide users with an optimal process parameter (LP, LS, SS and VP) combination ensuring required mechanical response.

However, prior to discussing FIS, ANN and ANFIS performance, it is necessary to explore the relationships (correlations) between the SLS parameters (LP, LS, SS and VP) and the mechanical properties (UTS, YM and EaB). This is done through the Pearson correlation analysis in the next sub-section.

### Correlation analysis

#### Pearson’s correlation analysis

As mentioned above, the Pearson correlation analysis was conducted to quantify the strength and direction of linear relationships between the variables. Pearson’s correlation coefficient, $r$, represents the degree of linear association between two sets of data, with values ranging from -1 to 1, where 0 meaning no linear correlation [42]. The calculated Pearson’s correlation coefficients for each parameter are presented in Table 3. According to the established guidelines [43], the strength of correlation coefficients can be classified based on their absolute values as: very weak correlation (0–0.2), weak correlation (0.2–0.4), moderate correlation (0.4–0.6), strong correlation (0.6–0.8) and very strong correlation (0.8–1).

**Table 3:** Pearson’s correlation coefficients for each SLS process parameters and mechanical characteristics

Feature	LP	LS	SS	VP	UTS	YM	EaB
LP	1.00	− 0.05	− 0.02	0.17	0.23	0.04	0.33
LS	− 0.05	1.00	0.02	0.05	− 0.44	− 0.42	− 0.54
SS	− 0.02	0.02	1.00	0.02	− 0.43	− 0.35	− 0.60
VP	0.17	0.05	0.02	1.00	− 0.10	− 0.06	0.09

#### SLS process parameters correlations

The first part of the analysis focuses on the relationship between the SLS process parameters. Understanding these correlations is crucial to identify potential multicollinearity and ensure that the selected SLS process features provide distinct and non-redundant information for the modelling. Key observations from the performed correlation analysis are summarised in Table 3: LP and LS exhibit a very weak negative correlation; SS shows very weak correlations with both LP and LS; VP shows very weak correlations with LP, SS and LS. These results indicate that all analysed parameters are largely independent and do not strongly influence each other within the given dataset.

These correlation coefficients suggest that there is minimal multicollinearity between analysed SLS process parameters, ensuring that each parameter provides distinct and meaningful information for the modelling process.

#### Correlations between SLS process parameters and mechanical characteristics

Understanding these relationships is critical for investigating which input SLS parameter has the strongest influence on the specific mechanical property and will validate the feature selection for the models. Key observations from the SLS process parameter-mechanical properties correlation analysis are presented in Table 3. To re-iterate: LP exhibits a very weak positive correlation with YM, and a weak positive correlation with UTS and EaB, indicating that higher LP can have an only minor impact on UTS, YM and EaB; LS shows a moderate negative correlation with UTS, YM and EaB, indicating that higher LS tends to reduce all three mechanical properties; SS shows a moderate negative correlation with UTS, YM and EaB, implying that with a higher SS, it is expected to observe a lower UTS, YM and EaB; VP shows a very weak negative correlation with UTS, YM and very weak positive correlation with EaB, indicating that it does not significantly affect these mechanical properties.

It is important to note that while a high correlation coefficient indicates a strong linear relationship between two parameters, it does not necessarily guarantee accurate estimations in a predictive model [44]. If the true relationship between variables is non-linear, a high linear correlation may fail to capture the underlying pattern. In this case, non-linear models, such as ANN, is needed to provide accurate predictions [45]. Additionally, a high correlation between two variables could be spurious as it can be caused by another unobserved variable [46].

Thus, while VP exhibits very weak correlation with mechanical characteristics, it could still interact with other input variables during the estimation process. Therefore, VP will be retained for further analysis in the subsequent modelling framework to investigate its potential interactions.

#### Pareto analysis for the SLS process parameters and mechanical characteristics

Building upon the above Pearson’s correlation analysis, a Pareto analysis [47] was conducted to further evaluate the relative significance of the process parameters (LP, LS, SS and VP) on the mechanical characteristics (UTS, YM and EaB). While the Pearson analysis provided insights into the relationships between parameters, the Pareto analysis consolidates this information into a ranked assessment of each parameter’s influence, offering a clearer perspective on their cumulative contributions.

To take an example of how the Pareto charts are created, the total contribution of LP, LS, SS and VP to UTS is calculated as 0.23 + 0.44 + 0.43 + 0.1 = 1.2, based on the coefficients in Table 3. Hence, following a descending order, the individual contribution of each process parameter can be calculated as:

LS (UTS) = (0.44/1.2) * 100 = 36.7%, SS (UTS) = (0.43/1.2) * 100% = 35.8%, 

LP (UTS) = (0.23/1.2) * 100 = 19.2%, VP (UTS) = (0.1/1.2) * 100% = 8.3%.

On the other hand, the cumulative contribution of each parameter can be expressed as:

LS = 36.7%, LS + SS = 36.7% + 35.8% = 72.5%,

LS + SS + LP = 72.5% + 19.2% = 91.7%, LS + SS + LP + VP = 91.7% + 8.3% = 100%.

**Fig. 2:**
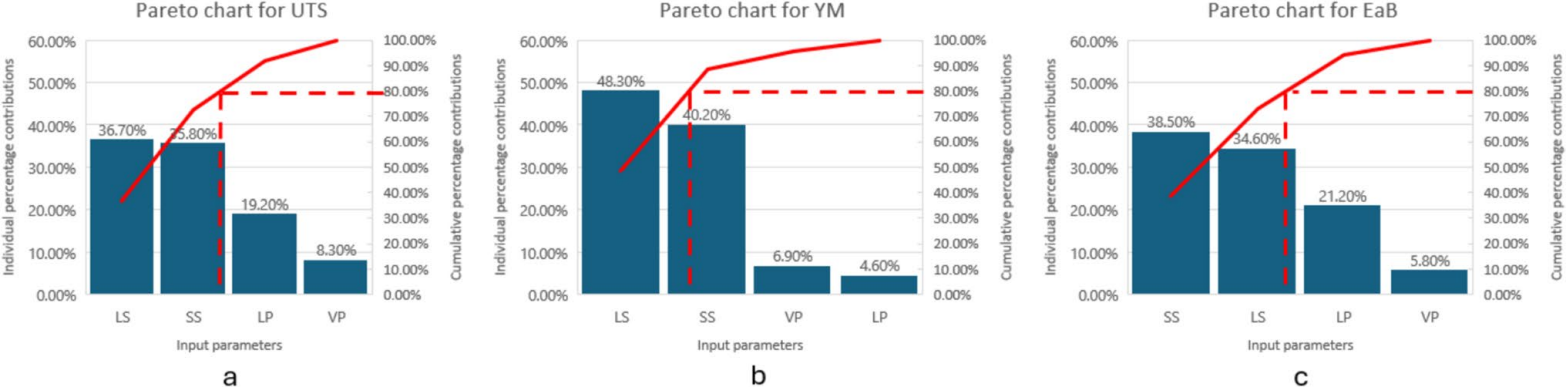
Pareto chart for assessing the significance of process parameters and **a** UTS, **b** YM, **c** EaB

The Pareto chart shown in Fig. 2 highlights notable trends in parameter significance. For UTS, LS (36.7%) and SS (35.8%) are the dominant contributors, jointly accounting for over 70% of the total contribution. Similarly, for YM, LS (48.3%) exhibits the most substantial influence, followed by SS (40.2%). In the case of EaB, SS (38.5%) and LS (34%) emerge as the leading factors, together covering over 70% of the impact. Notably, LP has a moderate impact, particularly on UTS and EaB, but is less critical for YM. Last but not least, the consistently small contribution of VP suggests its limited significance for all three mechanical characteristics, as previously suggested by the Pearson correlation analysis.

By identifying LS and SS as the primary contributors to mechanical characteristics, the Pareto analysis reinforces the findings from the Pearson analysis and serves as a foundation for the subsequent development of direct and inverse estimation frameworks.

### Direct estimation framework: from laser printer settings to mechanical properties of printed parts

In order to evaluate the performance of the three proposed data-driven methodologies, it is of great importance to have not only enough data to establish the “training” group for the model, but also a separate group of data for validation exclusively. To this end, each second of three samples is selected as a part of a validation set (shaded in Table 2), so that the training/validation conforms to the 70%/30% split, which is a generally accepted way to train and validate FIS models [48].

#### Fuzzy inference system predictions

As stated above, the idea of the direct framework is to predict mechanical properties of SLS parts, for known process parameters. The first methodology to be analysed is the fuzzy inference system. The unshaded rows in Table 2 are the training data, which are taken to build fuzzy rules; the input parameters of validation data (columns 2–5 in shaded rows in Table 2) are then fed into the formulated FIS. Unlike ANN and ANFIS methods, additional data scaling is unnecessary in FIS as membership functions essentially normalise variables into degrees of membership (values between 0 and 1).

The estimated results of FIS, ${E}_{est}$, are reported in Supplementary Table 1, together with observed experimental results, ${E}_{exp}$ (taken from columns 6–8 in shaded rows in Table 2). Supplementary Table 1 also reports the calculated percentage errors for each analysed mechanical characteristics, indicating the accuracy of the proposed techniques:

1 $$\begin{array}{c}PctE= \frac{\left|{E}_{est}-{E}_{exp}\right|}{{E}_{exp}} \times 100\%\end{array}$$

**Fig. 3:**
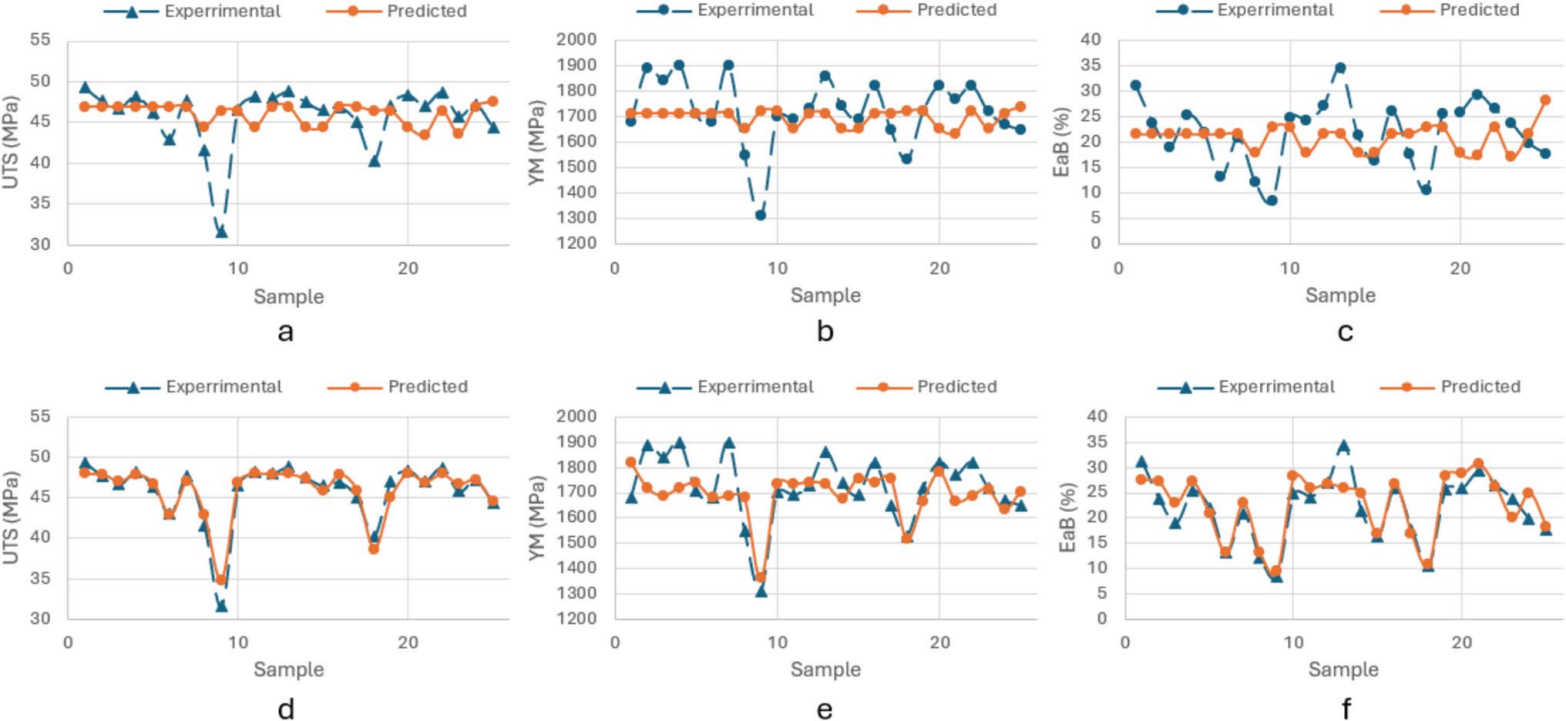
Comparison of experimental and predictive results for **a** UTS, **b** YM, **c** EaB using individual parameters, and **d**) UTS, **e** YM, **f** EaB using combined ED using FIS method

With FIS being applied within the direct framework, the reported average estimation errors are *6.16%*, *6.17%* and *32.79%* for UTS, YM and EaB respectively. Although the estimation accuracy for UTS and YM are good, 32.79% error for EaB is less satisfying due to some “outlier” points As presented in Fig. 3a–c, the predictive results using FIS method align relatively closely with the experimental results apart from a few notable outlier points.

It is, however, important to point out that although the FIS approach can estimate output parameters accurately in most cases, some large errors are still observed. More specifically, samples 26 and 53 have unacceptably high errors for all output parameters, compared to other samples.

It should be noted here, that one of the shortcomings of FIS, as mentioned above, is that this methodology may struggle when dealing with high-dimensional datasets, and may show less flexibility in adapting to complex, non-linear relationships [38]. As follows from Pearson’s correlation study, SLS process parameters and mechanical characteristics do not show very strong linear relationships, suggesting that the relations could potentially be non-linear. To this end models such as ANN and ANFIS are better candidates to provide accurate predictions [43].

However, prior to testing the performance of ANN and ANFIS, a possible alternative solution is proposed: to combine input parameters into a single feature parameter, such as the energy density. Creating a single “composite” feature could allow for capturing the combined effects of several input parameters into a single characteristic, which potentially could reduce the high error, and the additional complexity caused by the high dimensionality. This hypothesis will be tested in the section below.

#### Alternative input parameter: energy density

Analysis of the experimental results presented in Table 2 shows that some samples appear to have similar mechanical properties, however the combination of process parameters were different. More specifically, samples 50 and 74 both had similar YM—1650 MPa and EaB—17.7% and very close UTS—45 MPa and 44.4 MPa respectively, whereas the sets of process parameters for both samples were different (sample 50: LP—21, LS—3000, SS—0.25, VP—2; sample 74: LP—24, LS—3000, SS—0.3, VP—3). Such an ambiguous relationship can be found when various input parameter combinations lead to the same output parameter and could cause unexpected inaccuracy in determining the optimal process parameters. Thus, together with addressing the issue of added complexity, caused by the high dimensionality, on one hand, and potentially help resolving the non-uniqueness, on the other hand, we will argue the case of an alternative input parameter—energy density (ED) to combine the effects and replace single parameters LP, LS and SS.

In the application of SLS, ED is often used as a common parameter of interest, representing the energy transferred to the powder bed. It was found in previous studies that ED has a significant influence on physical and mechanical properties, dimensional accuracy as well as porosity of printed parts [49, 50]. Thus, it is of interest to introduce ED as an alternative process parameter in the current study. ED, having units ($\frac{J}{{mm}^{2}}$) by definition includes all previously considered process parameters (LP, SS and LS) except for the VP:

2 $$\begin{array}{c}ED= \frac{LP}{SS \times LS}\end{array}$$

Using samples 50 and 74 as examples, it can be seen that ED for both samples have similar calculated values: ED_50 = 0.028 $\frac{J}{{mm}^{2}}$ and ED_74 = 0.027 $\frac{J}{{mm}^{2}}$, albeit as a result of different combinations of the three original input parameters. Realising these similarities, the reported above closeness in mechanical properties, i.e. YM, UTS and EaB, seems now logical. Interesting to note that the Pearson’s correlation analysis has demonstrated higher degree of correlation between a single feature ED and all three mechanical properties (correlation coefficient between ED and UTS, YM and EaB was found to be 0.51, 0.39 and 0.78 respectively, i.e. moderate to strong correlation). With LP, SS and LS being combined into a single ED parameter, the new updated estimation results using the FIS algorithm are reported in Supplementary Table 2. Analysing these new predictions, it can be seen in Supplementary Table 2 that including alternative input parameter ED increases estimation accuracy: see errors of *1.72%*, *4.63%* and *9.51%* for UTS, YM and EaB respectively. Figure 3d–f also shows a closer alignment between experimental and predictive results when replacing individual parameters with a combined input parameter ED.

Interestingly, an increase in accuracy is reported for all three predicted mechanical properties, with a particular improvement in EaB, i.e. the estimation error decreased from *32.79%* to *9.70%*. This could be because combining LP, SS and LS with the single ED minimises the non-linearity in the original problem and thus, leads to an increasing estimation accuracy. Summarising, replacing LP, SS and LS with ED in the direct estimation framework can remove ambiguity on one hand and potentially lead to a good estimation result on the other.

Note that, since replacing LP, SS and LS with ED leads to a better estimation accuracy, the aforementioned three parameters will be replaced by ED in the following ANN and ANFIS direct framework sections for better estimation performance.

#### Artificial neural network predictions

One of the objectives of this paper is the comparative analysis of three data-driven methodologies (FIS, ANN and ANFIS), thus in this subsection artificial neural networks (ANN) is evaluated. For consistency, the selection of training and validation data remains the same as that of the FIS approach: 74 samples are tested in total and 25 of them have been classified as the validation group, whose outputs are assumed to be unknown. Following the logic of the FIS approach, the inputs of validation data are fed into the ANN, trained with 49 training samples, and the estimation results from the network are then compared with the actual experimental results. Based on the conclusion in the previous section, ED and VP have been considered here as input parameters.

As for the details of applied ANN model, specifically in this study, a back-propagation ANN is adopted with a gradient descent optimisation algorithm, as well as a commonly accepted learning rate of 0.01 [51]. There are two input variables—ED and VP, three output variables—UTS, YM and EaB. Additionally, there are three different layers—(i) an input layer, (ii) an output layer, and (iii) a hidden layer, as it has been proven that a single hidden layer is sufficient for predicting non-linear relationships in smaller datasets to prevent unnecessary complexity [52–54]. In order to prevent overfitting, which will significantly reduce the estimation accuracy, the number of neurons in the hidden layer is determined to be 5, considering the widely accepted guidelines [55–57]. In the hidden layer, the adopted activation function is the hyperbolic tangent sigmoid (Tansig) function, with benefit of faster convergence in training process [58] and being a common choice in shallow networks [58]. In the output layer, since the objective is to predict real numbers with few calculations, the applied activation function is pure linear function [31].

To ensure consistent and efficient training of the ANN model, both input and output variables are scaled prior to training using min–max scaling methods [59] and the value of variables are transformed to a normalised range of [0,1]. This ensures not only a faster convergence of the network, but also all features contribute equally to the training process and avoids dominance of variables with larger magnitudes over those with smaller ones. The scaled variables are calculated as:

3 $$\begin{array}{c}{x}{^{\prime}}=\frac{x-{x}_{min}}{{x}_{max}-{x}_{min}}\end{array}$$

where $x$ refers to the original variable, ${x}_{min}$ and ${x}_{max}$ are the minimum and maximum values of $x$ in the training dataset. After the prediction, the outputs are unscaled as follows to return to their original units for meaningful interpretation:

4 $$\begin{array}{c}y={y}{^{\prime}}\cdot \left({y}_{max}-{y}_{min}\right)+{y}_{min}\end{array}$$

where $y$ refers to the original meaningful output, ${y}_{min}$ and ${y}_{max}$ are the minimum and maximum values of $y$ and ${y}{\prime}$ is the standardised predicted output.

**Fig. 4:**
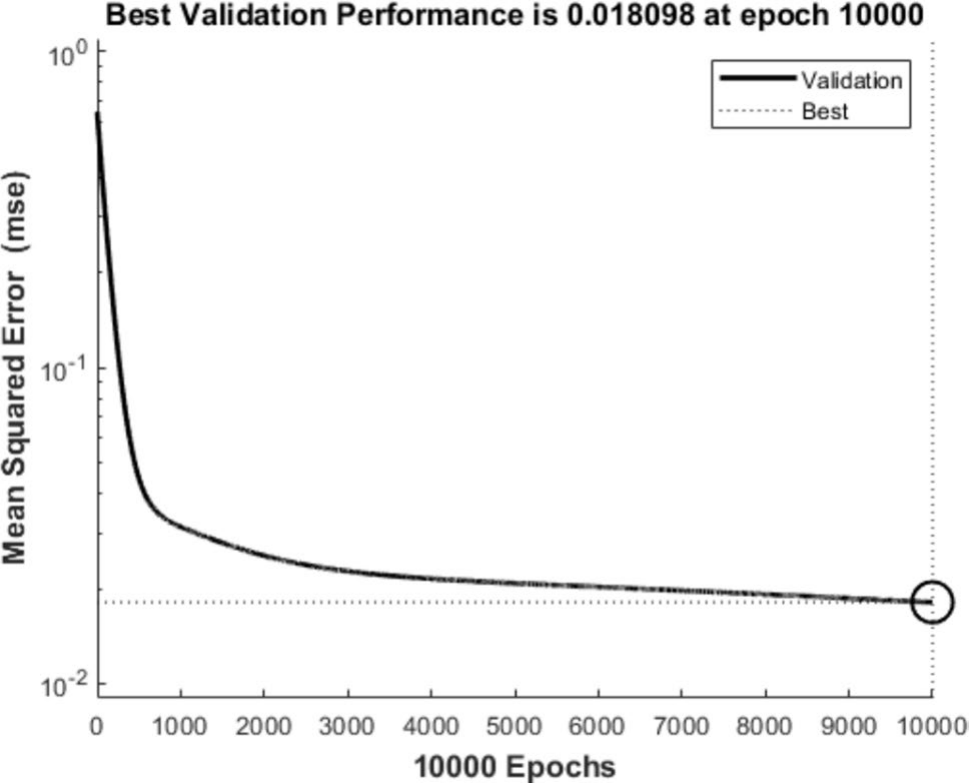
Mean squared error of the training process up to 10,000 epochs

As per the training process, Fig. 4 tracks the mean squared error (MSE) change over time and the estimation epoch is set to 10,000 after testing different epoch numbers. It is seen that the validation errors stabilised after approximately 6000 epochs and the least MSE is equal to 0.018098 at epoch 10,000. The MSE over time does not show large fluctuations or increasing, which indicates that overfitting is avoided and the model architecture is appropriate for good convergence.

**Fig. 5:**
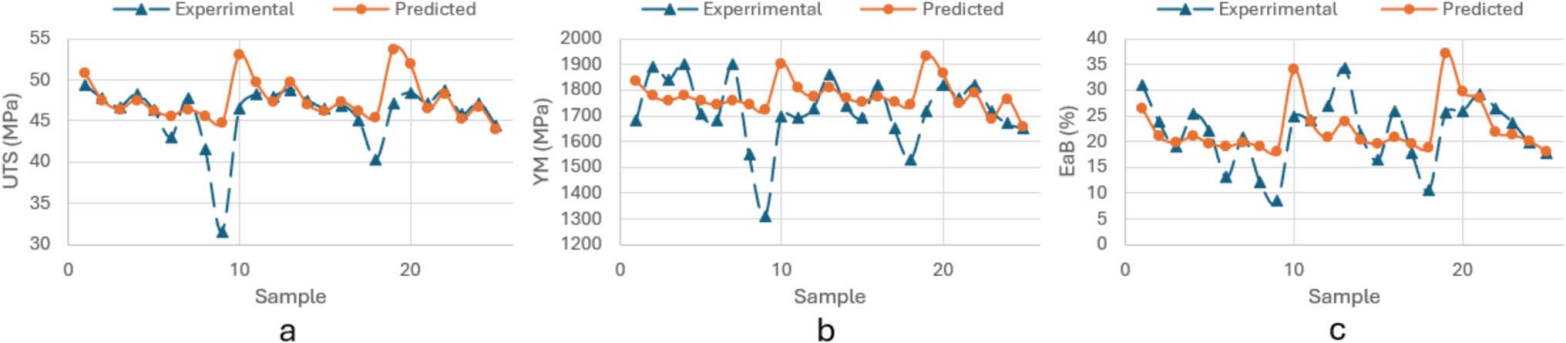
Comparison of experimental and predictive results for **a** UTS, **b** YM, **c** EaB using ANN method

The estimated and unscaled results using ANN and corresponding percentage errors PctEs are presented in Supplementary Table 3**:** The average estimation error, following ANN methodology, is reported as *5.21%* for UTS, *6.49%* for YM and *23.78%* for EaB, respectively. Combined with the close alignment between experimental and predictive results using ANN method shown in Fig. 5a–c, it can be concluded that ANN has shown the capability to estimate mechanical parameters based on provided processing parameters with good accuracy. It is seen that, similar to the results of the FIS approach, UTS has the best accuracy of predictions. With similar high estimation accuracy of UTS and YM, it is notable that ANN has resulted in larger estimation error for EaB (ANN—23.78%, FIS—9.7%). Such results will be compared further with the alternative data-driven approach (ANFIS).

#### Adaptive neural fuzzy inference system predictions

In this subsection, again the same experimental data was analysed using adaptive neural fuzzy inference system (ANFIS). Similar to previous analysis, 25 samples were classified as validation data and 49 samples were taken as a training group. Here once again ED and VP were taken as input parameters. The training data was first fed into the ANFIS to identify the parameters of the system and then the validation data was brought into the system for estimations. Notably, with an ANN-like structure, both input and output variables in the ANFIS section are scaled prior to training following the same method described in the ANN section.

For the adopted ANFIS model, the triangular MFs were chosen for both input variables, due to their computational efficiency and simplicity [60, 61]. There were three MFs used for each input variable, allowing for a good balance between model complexity and interpretability. A comparative analysis of estimation accuracy for cases of two, four and five MFs demonstrated that applying three MFs for each input variable is sufficient for capturing the non-linear relationship between input and output variables without overcomplicating the model [36]. Thus, there were $3\times 3=9$ fuzzy rules formulated in the model. Finally, the hybrid learning algorithm was used for training the ANFIS model, which combines least-squares estimation for optimising the consequent parameters and gradient descent for tuning the premise parameters, leading to faster convergence and more accurate estimation than using gradient descent alone [36].

**Fig. 6:**
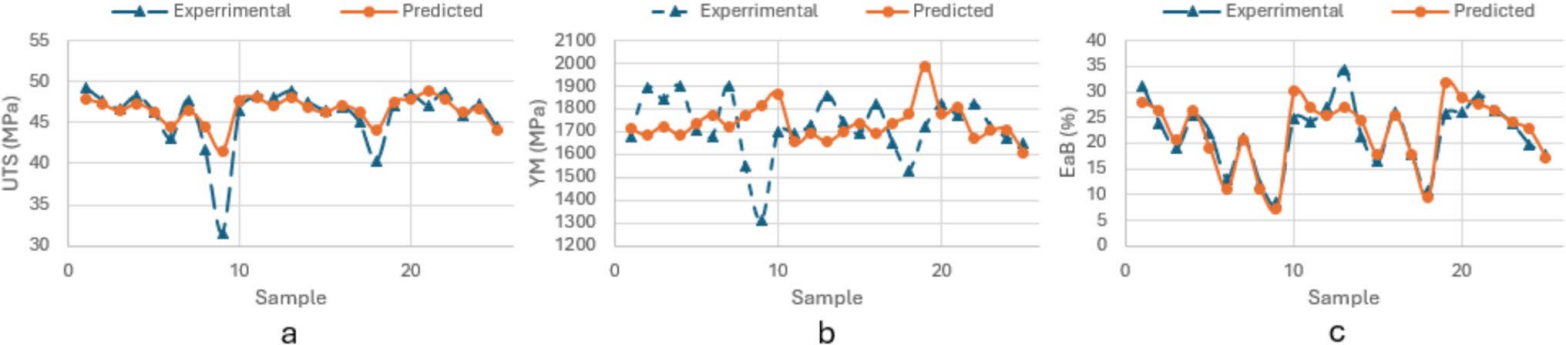
Comparison of experimental and predictive results for **a** UTS, **b** YM, **c** EaB using ANFIS method

The estimated (following ANFIS) outputs are again compared to the experimental results, and percentage errors as reported in Supplementary Table 4. It can be seen from Supplementary Table 4 that the average estimation error of using ANFIS approach is *3.27%* for UTS, *7.72%* for YM and *9.77%* for EaB respectively. With the presented comparative results in Fig. 6a–c using ANFIS method, ANFIS has shown a good estimation accuracy in the direct framework.

#### FIS, ANN and ANFIS comparative analysis of predictions accuracy

**Fig. 7:**
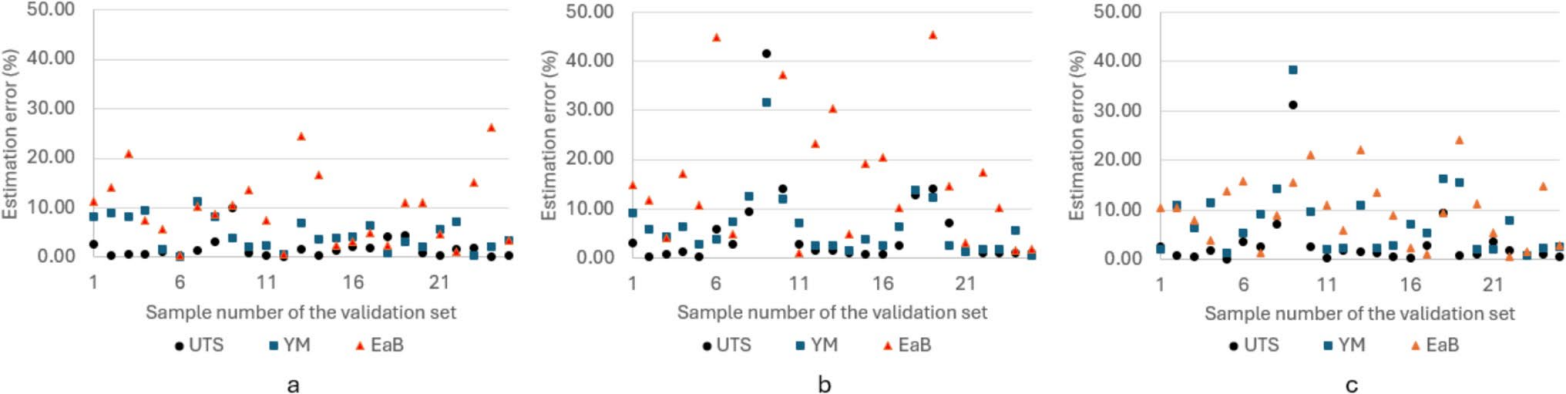
Direct estimation error for using **a** FIS, **b** ANN and **c** ANFIS

To summarise the results of all three approaches (FIS, ANN and ANFIS) and compare their performances, percentage errors of all three predicted mechanical characteristics (UTS, YM and EaB) were collected in Table 4. Figure 7 shows the comparative analysis of individual estimation error of using three different methodologies.

**Table 4:** Comparison of three methodologies in the direct estimation framework

	PctE UTS (%)	PctE YM (%)	PctE EaB (%)
FIS	1.72	4.63	9.51
ANN	5.21	6.49	23.78
ANFIS	3.27	7.72	9.77

Analysing the results presented in the table and the figure, it can be seen that although all three methodologies could offer good accuracy of predictions. In the current analysis for the direct framework, FIS is concluded to have the best performance, when compared with other two approaches.

### Inverse estimation framework: from desired mechanical properties of printed parts to laser printer settings

As previously mentioned, the goal of the inverse framework is to identify the optimal solution of SLS process parameters, ensuring the predetermined mechanical characteristics, i.e. UTS, YM and EaB. It has been previously reported [62], that the FIS approach has been successfully implemented in a similar type of inverse estimation framework. To formulate the inverse estimation framework, in the present investigation, UTS, YM and EaB have now been classified as inputs, and LP, LS, SS and VP as output parameters. For the consistency of the investigation, the training and validation data classification follows the same strategy adopted in the direct framework. 74 samples were tested in total, 49 of which were put in the training group and 25 samples formed the validation group. Data scaling methods prior to training for ANN and ANFIS follows the same procedure introduced in previous sections. The observed experimental results, together with the inversely estimated process parameters and the inverse estimation errors, are presented in Table 5 for all three methodologies and in Supplementary Table 5, Supplementary Table 6 and Supplementary Table 7 for using each methodology.

**Table 5:** Experimental and estimation results using FIS, ANN and ANFIS (inverse framework)

	Experimental results					PctE FIS					PctE ANN					PctE ANFIS				
Sample	LP (W)	LS (mm/s)	SS (mm)	VP	ED	LP (%)	LS (%)	SS (%)	VP (%)	ED (%)	LP (%)	LS (%)	SS (%)	VP (%)	ED (%)	LP (%)	LS (%)	SS (%)	VP (%)	ED (%)
2	18	2000	0.2	2	0.045	16.67	25	25	0	11.11	14.39	2.85	7.95	6.75	30.79	2.46	34.35	0.30	3.00	21.87
5	18	2000	0.25	2	0.036	16.67	0	0	50	16.67	11.28	13.31	10.25	9.39	1.34	6.43	8.65	0.20	6.20	3.00
8	18	2000	0.3	2	0.030	16.67	25	16.67	0	33.33	6.79	29.32	14.25	0.52	6.58	21.37	49.25	5.23	10.20	2.93
11	18	2500	0.2	2	0.036	16.67	0	25	0	11.11	12.96	12.32	10.82	6.07	9.25	9.13	13.24	15.40	21.00	14.56
14	18	2500	0.25	2	0.029	16.67	0	0	0	38.89	15.61	5.41	7.26	3.27	4.35	17.70	2.76	5.68	35.00	0.83
17	18	2500	0.3	2	0.024	0	0	0	50	0.00	6.63	12.28	8.02	1.73	9.53	1.04	9.96	9.90	6.90	1.67
20	18	3000	0.2	2	0.030	16.67	16.67	25	0	33.33	6.17	22.04	15.94	2.58	3.01	2.44	1.73	24.75	24.60	7.33
23	18	3000	0.25	2	0.024	16.67	0	20	50	2.78	6.95	7.48	9.55	7.24	13.31	2.65	8.77	17.92	84.80	8.83
26	18	3000	0.3	2	0.020	8.46	0	0	50	8.46	7.43	0.67	0.53	21.44	20.71	4.47	3.47	2.03	6.60	1.53
29	21	2000	0.2	2	0.053	0	25	25	0	23.81	1.71	24.28	25.20	8.22	28.16	2.43	23.95	31.25	28.80	26.48
32	21	2000	0.25	2	0.042	0	25	0	0	4.76	0.19	21.40	2.45	5.33	15.77	6.77	24.40	8.64	21.80	19.62
35	21	2000	0.3	2	0.035	14.29	0	16.67	50	2.86	0.68	12.40	23.43	0.65	30.89	1.29	20.65	22.87	2.10	11.20
38	21	2500	0.2	2	0.042	0	0	25	0	4.76	1.95	17.45	8.65	4.33	36.62	22.86	19.86	24.55	1.40	6.76
41	21	2500	0.25	2	0.034	0	0	0	0	19.05	2.78	2.17	6.07	3.44	13.37	0.17	2.48	6.12	11.40	10.95
44	21	2500	0.3	2	0.028	0	0	16.67	0	42.86	8.83	8.00	7.79	9.51	14.86	11.62	4.16	3.97	60.80	3.71
47	21	3000	0.2	2	0.035	0	16.67	25	0	14.29	0.66	22.61	13.31	14.07	16.94	6.40	25.57	21.20	33.60	15.66
50	21	3000	0.25	2	0.028	14.29	16.67	0	50	2.86	6.63	8.82	10.37	2.46	15.63	16.61	9.17	2.72	24.50	2.57
53	21	3000	0.3	2	0.023	0	16.67	16.67	0	71.43	8.45	6.08	7.73	8.46	11.09	2.74	3.10	10.00	48.25	3.20
56	24	2000	0.2	2	0.060	4.38	25	8.76	14.95	28.41	11.92	19.16	20.08	6.23	32.59	11.50	21.15	24.70	16.10	35.00
59	24	2000	0.25	2	0.048	12.5	25	0	0	16.67	14.57	9.64	9.78	0.95	16.37	15.05	14.10	5.04	22.90	16.75
62	24	2000	0.3	3	0.040	12.5	25	16.67	33.33	0.00	11.88	11.93	26.49	26.78	26.98	15.18	14.80	33.48	12.67	7.40
65	24	2500	0.25	2	0.038	12.5	0	0	0	4.17	14.75	13.98	10.45	1.89	8.48	12.65	9.00	5.76	29.80	5.83
68	24	2500	0.3	3	0.032	25	20	33.33	0	6.25	11.87	2.57	14.93	25.30	7.20	12.65	1.24	11.33	2.00	11.13
71	24	3000	0.25	2	0.032	12.5	16.67	0	0	25.00	15.33	11.42	11.59	12.77	18.55	7.03	16.03	6.28	7.40	9.63
74	24	3000	0.3	3	0.027	25	16.67	16.67	0	8.00	18.23	8.49	8.37	32.86	11.21	27.11	5.37	16.47	1.67	2.15
	Average Pct error					10.32	11.8	12.48	13.93	17.23	8.75	12.24	11.65	8.89	16.14	9.59	13.89	12.63	20.94	10.2

**Fig. 8:**
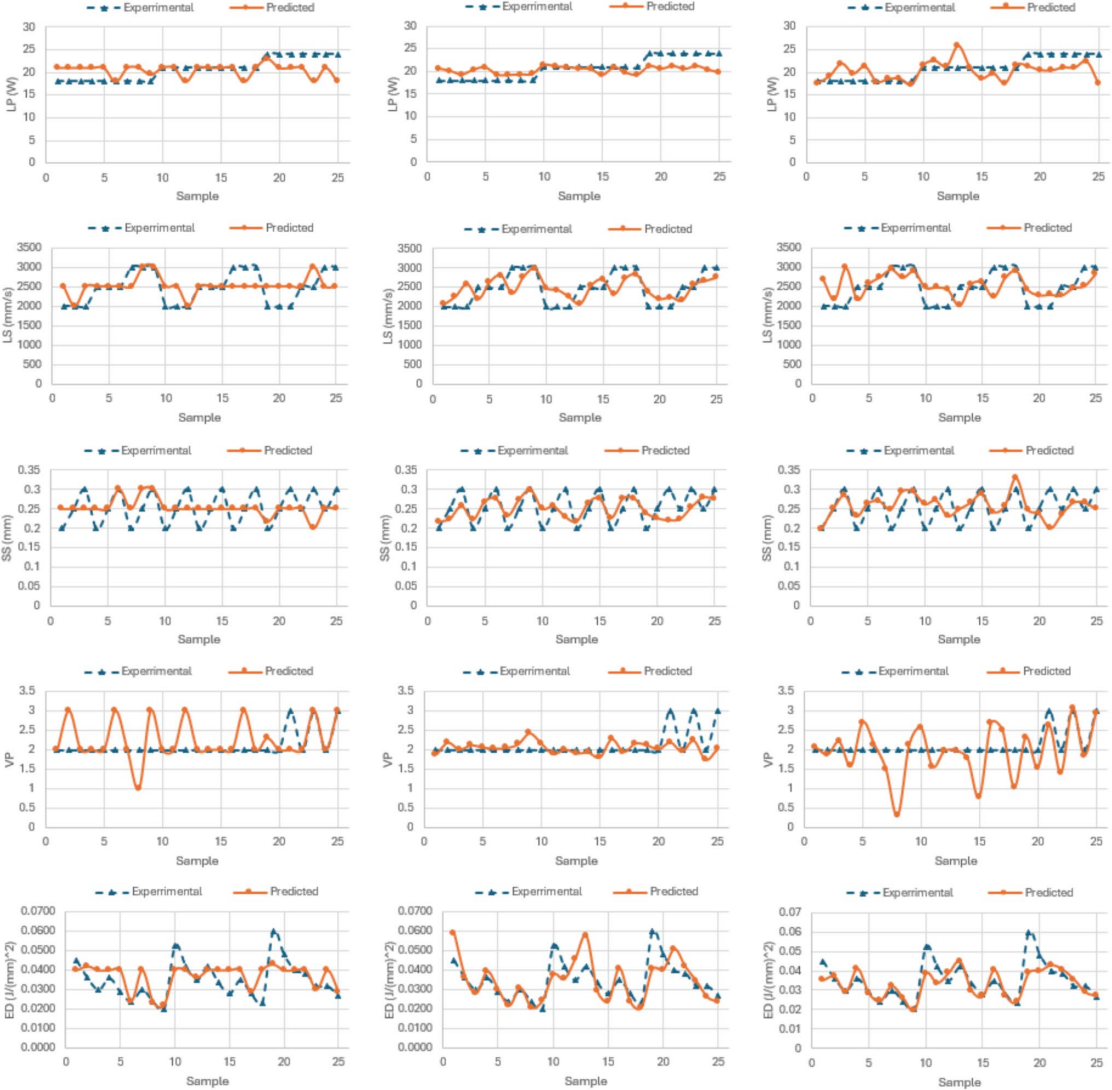
Comparison of inverse estimation results using FIS (left column), ANN (middle column) and ANFIS (right column) methods to predict LP (first row), LS (second row), SS (third row), VP (fourth row) and ED LP (fifth row)

As it can be seen from Table 5, all three data-driven methodologies have reasonable estimation accuracy also in the inverse framework. Analysing the comparative results, for FIS approach, the average estimation error is *10.32%* for LP, *11.8%* for LS, *12.48%* for SS, *13.93%* for VP and 17.23% for ED respectively. As for ANN approach, the average error is reported to be *8.75%* for LP, *12.24%* for LS, *11.65%* for SS, *8.89%* for VP and 16.14% for ED respectively. Finally for the ANFIS approach, the average error is *9.59%* for LP, *13.89%* for LS, *12.63%* for SS, *20.94%* for VP and 10.2% for ED respectively. Interesting to note, that although all process parameters can be fairly accurately estimated, the LP can be estimated with the best accuracy in the inverse framework. Given the previously mentioned weak correlation between VP and mechanical properties, the misalignment observed in the fourth row of Fig. 8 is unsurprising.

Bringing together the aforementioned results with the comparative experimental and predictive results presented in Fig. 8, it can be concluded that ANN methodology could potentially offer more stable and accurate predictions in the inverse estimation framework for the analysed problem.

Generally, it can be seen that the direct estimation has better accuracy of predictions than the inverse framework. For the SLS, process parameters are the “premise” of mechanical properties, which as a “consequence” can be also affected by other “premises”, for example, material related and pre/post processing related parameters. Thus, the slight accuracy decrease in the inverse estimation is reasonable and foreseeable.

## Conclusion

The main aim of the present investigation was to evaluate the performance of data-driven methodologies to formulate relationships between various process parameters and the mechanical properties of SLS printed components. Based on the above analysis and discussion, fuzzy inference system (FIS), artificial neural networks (ANN) and adaptive neural fuzzy inference system (ANFIS) have shown their capabilities in the prediction of UTS, YM and EaB, with provided laser settings—LP, LS and SS, or their combined effect, ED, along with the VP. On the other hand, data-driven techniques have also shown their abilities to reliably predict laser settings based on above desired mechanical properties, i.e. an inverse framework, which is potentially more relevant in industrial applications.

The results presented in the paper indicate that all three considered data-driven approaches have a high degree of accuracy in their predictions. Interesting to note that for the direct framework, FIS approach provides the most accurate estimation results, compared with ANN and ANFIS method. While in the inverse framework, ANN has a better estimation accuracy.

## Supplementary Information

Below is the link to the electronic supplementary material.

Supplementary file1 (DOCX 60 KB)

## Data Availability

All data generated or analysed during this study are included in this article and its supplementary information files.

## Abbreviations

*E*_*est*_: Estimated parameter
*E*_*exp*_: Experimental parameter
PctE: Estimation error in percentage
LP: Laser power
LS: Laser speed
SS: Scan spacing
VP: Vertical position
UTS: Ultimate tensile strength
YM: Young’s modulus
EaB: Elongation at break
