# Supplementary material for: Data-driven approaches for predicting mechanical properties and determining processing parameters of selective laser sintered nylon-12 components
Source: Discov Mech Eng. 2025 Mar 22;4(1):10. doi: 10.1007/s44245-025-00094-7 (PMC11929718; doi:10.1007/s44245-025-00094-7)
Supplement: Supplementary file 1 — Supplementary file1 (DOCX 60 KB) [file 44245_2025_94_MOESM1_ESM.docx]

**Supplementary Table 1** Experimental and estimation results for using FIS in the direct framework

|  | Experimental results | | | Estimation results | | | Percentage Errors | | |
| --- | --- | --- | --- | --- | --- | --- | --- | --- | --- |
| Sample | UTS (MPa) | YM (MPa) | EaB (%) | Pred UTS (MPa) | Pred YM (MPa) | Pred EaB (%) | PctE UTS (%) | PctE YM (%) | PctE EaB (%) |
| 2 | 49.3 | 1680 | 31.1 | 46.96 | 1712.74 | 21.65 | 4.75 | 1.95 | 30.38 |
| 5 | 47.7 | 1890 | 23.8 | 46.96 | 1712.74 | 21.65 | 1.56 | 9.38 | 9.02 |
| 8 | 46.7 | 1840 | 19 | 46.96 | 1712.74 | 21.65 | 0.55 | 6.92 | 13.96 |
| 11 | 48.2 | 1900 | 25.3 | 46.96 | 1712.74 | 21.65 | 2.58 | 9.86 | 14.42 |
| 14 | 46.3 | 1710 | 22 | 46.96 | 1712.74 | 21.65 | 1.42 | 0.16 | 1.58 |
| 17 | 43 | 1680 | 13.1 | 46.96 | 1712.74 | 21.65 | 9.20 | 1.95 | 65.28 |
| 20 | 47.7 | 1900 | 20.8 | 46.96 | 1712.74 | 21.65 | 1.56 | 9.86 | 4.10 |
| 23 | 41.6 | 1550 | 12.1 | 44.44 | 1651.04 | 17.85 | 6.84 | 6.52 | 47.53 |
| 26 | 31.6 | 1310 | 8.5 | 46.43 | 1720.90 | 22.99 | 46.95 | 31.37 | 170.48 |
| 29 | 46.5 | 1700 | 24.8 | 46.43 | 1720.90 | 22.99 | 0.14 | 1.23 | 7.30 |
| 32 | 48.2 | 1690 | 24.2 | 44.44 | 1651.04 | 17.85 | 7.79 | 2.31 | 26.24 |
| 35 | 48 | 1730 | 27 | 46.96 | 1712.74 | 21.65 | 2.17 | 1.00 | 19.81 |
| 38 | 48.8 | 1860 | 34.4 | 46.96 | 1712.74 | 21.65 | 3.78 | 7.92 | 37.06 |
| 41 | 47.5 | 1740 | 21.4 | 44.44 | 1651.04 | 17.85 | 6.43 | 5.11 | 16.59 |
| 44 | 46.5 | 1690 | 16.4 | 44.44 | 1651.04 | 17.85 | 4.42 | 2.31 | 8.85 |
| 47 | 46.9 | 1820 | 26 | 46.96 | 1712.74 | 21.65 | 0.12 | 5.89 | 16.72 |
| 50 | 45 | 1650 | 17.7 | 46.96 | 1712.74 | 21.65 | 4.35 | 3.80 | 22.33 |
| 53 | 40.3 | 1530 | 10.6 | 46.43 | 1720.90 | 22.99 | 15.22 | 12.48 | 116.89 |
| 56 | 47.1 | 1720 | 25.6 | 46.43 | 1720.90 | 22.99 | 1.41 | 0.05 | 10.19 |
| 59 | 48.4 | 1820 | 25.9 | 44.44 | 1651.04 | 17.85 | 8.17 | 9.28 | 31.08 |
| 62 | 47.1 | 1770 | 29.3 | 43.48 | 1634.71 | 17.45 | 7.69 | 7.64 | 40.45 |
| 65 | 48.7 | 1820 | 26.5 | 46.43 | 1720.90 | 22.99 | 4.65 | 5.45 | 13.24 |
| 68 | 45.8 | 1720 | 23.7 | 43.64 | 1653.13 | 17.09 | 4.71 | 3.89 | 27.88 |
| 71 | 47.2 | 1670 | 19.8 | 46.96 | 1712.74 | 21.65 | 0.52 | 2.56 | 9.35 |
| 74 | 44.4 | 1650 | 17.7 | 47.56 | 1738.34 | 28.15 | 7.12 | 5.35 | 59.04 |
|  | | | | | | **Average** | **6.16** | **6.17** | **32.79** |

**Supplementary Table 2** Experimental and estimation results using FIS: ED as an alternative parameter (direct framework)

| Sample | Input parameters | | Percentage errors | | |
| --- | --- | --- | --- | --- | --- |
|  | ED ($\frac{J}{{mm}^{2}}$) | VP | PctE UTS (%) | PctE YM (%) | PctE EaB (%) |
| 2 | 0.0450 | 2 | 0.50 | 3.69 | 16.06 |
| 5 | 0.0360 | 2 | 0.47 | 8.99 | 14.15 |
| 8 | 0.0300 | 2 | 0.74 | 8.28 | 20.84 |
| 11 | 0.0360 | 2 | 0.58 | 9.47 | 7.38 |
| 14 | 0.0288 | 2 | 1.02 | 1.58 | 5.59 |
| 17 | 0.0240 | 2 | 0.30 | 0.05 | 0.46 |
| 20 | 0.0300 | 2 | 1.37 | 11.18 | 10.38 |
| 23 | 0.0240 | 2 | 3.06 | 8.34 | 8.77 |
| 26 | 0.0200 | 2 | 10.13 | 4.01 | 10.59 |
| 29 | 0.0525 | 2 | 0.89 | 2.17 | 13.67 |
| 32 | 0.0420 | 2 | 0.49 | 2.50 | 7.37 |
| 35 | 0.0350 | 2 | 0.19 | 0.69 | 0.74 |
| 38 | 0.0420 | 2 | 1.72 | 6.87 | 24.47 |
| 41 | 0.0336 | 2 | 0.42 | 3.60 | 16.58 |
| 44 | 0.0280 | 2 | 1.31 | 3.96 | 2.51 |
| 47 | 0.0350 | 2 | 2.15 | 4.29 | 3.08 |
| 50 | 0.0280 | 2 | 1.98 | 6.48 | 5.02 |
| 53 | 0.0233 | 2 | 4.06 | 0.82 | 2.36 |
| 56 | 0.0600 | 2 | 4.35 | 3.05 | 11.04 |
| 59 | 0.0480 | 2 | 0.86 | 2.20 | 11.13 |
| 62 | 0.0400 | 3 | 0.34 | 5.77 | 4.77 |
| 65 | 0.0384 | 2 | 1.53 | 7.25 | 1.11 |
| 68 | 0.0320 | 3 | 1.95 | 0.35 | 15.14 |
| 71 | 0.0320 | 2 | 0.00 | 2.27 | 26.12 |
| 74 | 0.0267 | 3 | 0.28 | 3.29 | 3.31 |
|  | | Average | **1.63** | **4.45** | **9.70** |

**Supplementary Table 3** Experimental and estimation results using ANN (direct framework)

|  | Experimental results | | | Estimation results | | | Percentage errors | | |
| --- | --- | --- | --- | --- | --- | --- | --- | --- | --- |
| Sample | UTS (MPa) | YM (MPa) | EaB (%) | Pred UTS (MPa) | Pred YM (MPa) | Pred EaB (%) | PctE UTS (%) | PctE YM (%) | PctE EaB (%) |
| 2 | 49.3 | 1680 | 31.1 | 50.81 | 1836.45 | 26.53 | 3.06 | 9.31 | 14.69 |
| 5 | 47.7 | 1890 | 23.8 | 47.52 | 1777.11 | 21.00 | 0.37 | 5.97 | 11.77 |
| 8 | 46.7 | 1840 | 19 | 46.37 | 1759.93 | 19.78 | 0.70 | 4.35 | 4.11 |
| 11 | 48.2 | 1900 | 25.3 | 47.52 | 1777.11 | 21.00 | 1.40 | 6.47 | 17.00 |
| 14 | 46.3 | 1710 | 22 | 46.22 | 1757.22 | 19.62 | 0.18 | 2.76 | 10.80 |
| 17 | 43 | 1680 | 13.1 | 45.57 | 1743.87 | 18.96 | 5.98 | 3.80 | 44.75 |
| 20 | 47.7 | 1900 | 20.8 | 46.37 | 1759.93 | 19.78 | 2.78 | 7.37 | 4.90 |
| 23 | 41.6 | 1550 | 12.1 | 45.57 | 1743.87 | 18.96 | 9.55 | 12.51 | 56.71 |
| 26 | 31.6 | 1310 | 8.5 | 44.70 | 1722.64 | 18.02 | 41.47 | 31.50 | 111.99 |
| 29 | 46.5 | 1700 | 24.8 | 52.99 | 1903.19 | 34.03 | 13.96 | 11.95 | 37.23 |
| 32 | 48.2 | 1690 | 24.2 | 49.61 | 1811.08 | 23.98 | 2.93 | 7.16 | 0.92 |
| 35 | 48 | 1730 | 27 | 47.27 | 1773.43 | 20.71 | 1.52 | 2.51 | 23.28 |
| 38 | 48.8 | 1860 | 34.4 | 49.61 | 1811.08 | 23.98 | 1.67 | 2.63 | 30.30 |
| 41 | 47.5 | 1740 | 21.4 | 46.96 | 1768.97 | 20.38 | 1.13 | 1.67 | 4.75 |
| 44 | 46.5 | 1690 | 16.4 | 46.12 | 1755.38 | 19.52 | 0.83 | 3.87 | 19.04 |
| 47 | 46.9 | 1820 | 26 | 47.27 | 1773.43 | 20.71 | 0.79 | 2.56 | 20.33 |
| 50 | 45 | 1650 | 17.7 | 46.12 | 1755.38 | 19.52 | 2.48 | 6.39 | 10.29 |
| 53 | 40.3 | 1530 | 10.6 | 45.46 | 1741.27 | 18.84 | 12.81 | 13.81 | 77.77 |
| 56 | 47.1 | 1720 | 25.6 | 53.73 | 1930.30 | 37.19 | 14.07 | 12.23 | 45.28 |
| 59 | 48.4 | 1820 | 25.9 | 51.87 | 1865.28 | 29.68 | 7.18 | 2.49 | 14.58 |
| 62 | 47.1 | 1770 | 29.3 | 46.49 | 1749.29 | 28.38 | 1.28 | 1.17 | 3.14 |
| 65 | 48.7 | 1820 | 26.5 | 48.25 | 1788.05 | 21.90 | 0.91 | 1.76 | 17.38 |
| 68 | 45.8 | 1720 | 23.7 | 45.27 | 1689.80 | 21.25 | 1.15 | 1.76 | 10.32 |
| 71 | 47.2 | 1670 | 19.8 | 46.67 | 1764.65 | 20.08 | 1.12 | 5.67 | 1.42 |
| 74 | 44.4 | 1650 | 17.7 | 43.98 | 1658.41 | 18.00 | 0.95 | 0.51 | 1.70 |
|  | | | | | | **Average** | **5.21** | **6.49** | **23.78** |

**Supplementary Table 4** Experimental and estimation results using ANFIS (direct framework)

|  | Experimental results | | | | | Estimation results | | | | Percentage errors | | |
| --- | --- | --- | --- | --- | --- | --- | --- | --- | --- | --- | --- | --- |
| Sample | | UTS (MPa) | YM (MPa) | EaB (%) | Pred UTS (MPa) | | Pred YM (MPa) | Pred EaB (%) | PctE UTS (%) | | PctE YM (%) | PctE EaB (%) |
| 2 | | 49.3 | 1680 | 31.1 | 47.94 | | 1714.60 | 27.85 | 2.76 | | 2.06 | 10.44 |
| 5 | | 47.7 | 1890 | 23.8 | 47.32 | | 1683.10 | 26.31 | 0.81 | | 10.95 | 10.53 |
| 8 | | 46.7 | 1840 | 19 | 46.43 | | 1723.00 | 20.50 | 0.57 | | 6.36 | 7.89 |
| 11 | | 48.2 | 1900 | 25.3 | 47.32 | | 1683.10 | 26.31 | 1.83 | | 11.42 | 3.98 |
| 14 | | 46.3 | 1710 | 22 | 46.32 | | 1732.10 | 18.98 | 0.05 | | 1.29 | 13.74 |
| 17 | | 43 | 1680 | 13.1 | 44.54 | | 1772.70 | 11.03 | 3.58 | | 5.52 | 15.82 |
| 20 | | 47.7 | 1900 | 20.8 | 46.43 | | 1723.00 | 20.50 | 2.66 | | 9.32 | 1.44 |
| 23 | | 41.6 | 1550 | 12.1 | 44.54 | | 1772.70 | 11.03 | 7.06 | | 14.37 | 8.86 |
| 26 | | 31.6 | 1310 | 8.5 | 41.50 | | 1812.60 | 7.18 | 31.33 | | 38.37 | 15.49 |
| 29 | | 46.5 | 1700 | 24.8 | 47.72 | | 1867.20 | 30.04 | 2.63 | | 9.84 | 21.14 |
| 32 | | 48.2 | 1690 | 24.2 | 48.03 | | 1653.70 | 26.83 | 0.35 | | 2.15 | 10.87 |
| 35 | | 48 | 1730 | 27 | 47.11 | | 1689.40 | 25.40 | 1.85 | | 2.35 | 5.92 |
| 38 | | 48.8 | 1860 | 34.4 | 48.03 | | 1653.70 | 26.83 | 1.57 | | 11.09 | 22.00 |
| 41 | | 47.5 | 1740 | 21.4 | 46.87 | | 1698.50 | 24.28 | 1.32 | | 2.39 | 13.48 |
| 44 | | 46.5 | 1690 | 16.4 | 46.27 | | 1738.40 | 17.88 | 0.50 | | 2.86 | 9.04 |
| 47 | | 46.9 | 1820 | 26 | 47.11 | | 1689.40 | 25.40 | 0.46 | | 7.18 | 2.30 |
| 50 | | 45 | 1650 | 17.7 | 46.27 | | 1738.40 | 17.88 | 2.82 | | 5.36 | 1.03 |
| 53 | | 40.3 | 1530 | 10.6 | 44.10 | | 1779.00 | 9.59 | 9.42 | | 16.27 | 9.53 |
| 56 | | 47.1 | 1720 | 25.6 | 47.54 | | 1989.00 | 31.80 | 0.93 | | 15.64 | 24.24 |
| 59 | | 48.4 | 1820 | 25.9 | 47.85 | | 1779.70 | 28.81 | 1.14 | | 2.21 | 11.22 |
| 62 | | 47.1 | 1770 | 29.3 | 48.77 | | 1806.30 | 27.69 | 3.54 | | 2.05 | 5.50 |
| 65 | | 48.7 | 1820 | 26.5 | 47.81 | | 1673.30 | 26.31 | 1.82 | | 8.06 | 0.73 |
| 68 | | 45.8 | 1720 | 23.7 | 46.23 | | 1703.40 | 24.07 | 0.94 | | 0.97 | 1.56 |
| 71 | | 47.2 | 1670 | 19.8 | 46.65 | | 1709.00 | 22.71 | 1.16 | | 2.34 | 14.71 |
| 74 | | 44.4 | 1650 | 17.7 | 44.11 | | 1608.20 | 17.19 | 0.64 | | 2.53 | 2.87 |
|  | | | | | | | | **Average** | **3.27** | | **7.72** | **9.77** |

**Supplementary Table 5** Experimental and estimation results using FIS (inverse framework)

|  | Experimental results | | | | Estimation results | | | | Percentage errors | | | |
| --- | --- | --- | --- | --- | --- | --- | --- | --- | --- | --- | --- | --- |
| Sample | Laser power (W) | Laser speed (mm/s) | Scan spacing (mm) | Vertical Position | Laser Power | Laser Speed | Scan spacing | Vertical Position | Laser Power (%) | Laser Speed (%) | Scan spacing (%) | Vertical Position (%) |
| 2 | 18 | 2000 | 0.2 | 2 | 21 | 2500 | 0.25 | 2 | 16.67 | 25 | 25 | 0 |
| 5 | 18 | 2000 | 0.25 | 2 | 21 | 2000 | 0.25 | 3 | 16.67 | 0 | 0 | 50 |
| 8 | 18 | 2000 | 0.3 | 2 | 21 | 2500 | 0.25 | 2 | 16.67 | 25 | 16.67 | 0 |
| 11 | 18 | 2500 | 0.2 | 2 | 21 | 2500 | 0.25 | 2 | 16.67 | 0 | 25 | 0 |
| 14 | 18 | 2500 | 0.25 | 2 | 21 | 2500 | 0.25 | 2 | 16.67 | 0 | 0 | 0 |
| 17 | 18 | 2500 | 0.3 | 2 | 18 | 2500 | 0.3 | 3 | 0 | 0 | 0 | 50 |
| 20 | 18 | 3000 | 0.2 | 2 | 21 | 2500 | 0.25 | 2 | 16.67 | 16.67 | 25 | 0 |
| 23 | 18 | 3000 | 0.25 | 2 | 21 | 3000 | 0.3 | 1 | 16.67 | 0 | 20 | 50 |
| 26 | 18 | 3000 | 0.3 | 2 | 19.52 | 3000 | 0.3 | 3 | 8.46 | 0 | 0 | 50 |
| 29 | 21 | 2000 | 0.2 | 2 | 21 | 2500 | 0.25 | 2 | 0 | 25 | 25 | 0 |
| 32 | 21 | 2000 | 0.25 | 2 | 21 | 2500 | 0.25 | 2 | 0 | 25 | 0 | 0 |
| 35 | 21 | 2000 | 0.3 | 2 | 18 | 2000 | 0.25 | 3 | 14.29 | 0 | 16.67 | 50 |
| 38 | 21 | 2500 | 0.2 | 2 | 21 | 2500 | 0.25 | 2 | 0 | 0 | 25 | 0 |
| 41 | 21 | 2500 | 0.25 | 2 | 21 | 2500 | 0.25 | 2 | 0 | 0 | 0 | 0 |
| 44 | 21 | 2500 | 0.3 | 2 | 21 | 2500 | 0.25 | 2 | 0 | 0 | 16.67 | 0 |
| 47 | 21 | 3000 | 0.2 | 2 | 21 | 2500 | 0.25 | 2 | 0 | 16.67 | 25 | 0 |
| 50 | 21 | 3000 | 0.25 | 2 | 18 | 2500 | 0.25 | 3 | 14.29 | 16.67 | 0 | 50 |
| 53 | 21 | 3000 | 0.3 | 2 | 21 | 2500 | 0.25 | 2 | 0 | 16.67 | 16.67 | 0 |
| 56 | 24 | 2000 | 0.2 | 2 | 22.95 | 2500 | 0.22 | 2.30 | 4.38 | 25 | 8.76 | 14.95 |
| 59 | 24 | 2000 | 0.25 | 2 | 21 | 2500 | 0.25 | 2 | 12.5 | 25 | 0 | 0 |
| 62 | 24 | 2000 | 0.3 | 3 | 21 | 2500 | 0.25 | 2 | 12.5 | 25 | 16.67 | 33.33 |
| 65 | 24 | 2500 | 0.25 | 2 | 21 | 2500 | 0.25 | 2 | 12.5 | 0 | 0 | 0 |
| 68 | 24 | 2500 | 0.3 | 3 | 18 | 3000 | 0.2 | 3 | 25 | 20 | 33.33 | 0 |
| 71 | 24 | 3000 | 0.25 | 2 | 21 | 2500 | 0.25 | 2 | 12.5 | 16.67 | 0 | 0 |
| 74 | 24 | 3000 | 0.3 | 3 | 18 | 2500 | 0.25 | 3 | 25 | 16.67 | 16.67 | 0 |
|  | | | | | | | | **Average** | **10.32** | **11.80** | **12.48** | **13.93** |

**Supplementary Table 6** Experimental and estimation results using ANN (inverse framework)

|  | Experimental results | | | | Estimation results | | | | Percentage errors | | | |
| --- | --- | --- | --- | --- | --- | --- | --- | --- | --- | --- | --- | --- |
| Sample | Laser power (W) | Laser speed (mm/s) | Scan spacing (mm) | Vertical Position | Laser Power | Laser Speed | Scan spacing | Vertical Position | Laser Power (%) | Laser Speed (%) | Scan spacing (%) | Vertical Position (%) |
| 2 | 18 | 2000 | 0.2 | 2 | 20.59 | 2057.00 | 0.22 | 1.86 | 14.39 | 2.85 | 7.95 | 6.75 |
| 5 | 18 | 2000 | 0.25 | 2 | 20.03 | 2266.30 | 0.22 | 2.19 | 11.28 | 13.31 | 10.25 | 9.39 |
| 8 | 18 | 2000 | 0.3 | 2 | 19.22 | 2586.45 | 0.26 | 2.01 | 6.79 | 29.32 | 14.25 | 0.52 |
| 11 | 18 | 2500 | 0.2 | 2 | 20.33 | 2191.94 | 0.22 | 2.12 | 12.96 | 12.32 | 10.82 | 6.07 |
| 14 | 18 | 2500 | 0.25 | 2 | 20.81 | 2635.26 | 0.27 | 2.07 | 15.61 | 5.41 | 7.26 | 3.27 |
| 17 | 18 | 2500 | 0.3 | 2 | 19.19 | 2806.94 | 0.28 | 2.03 | 6.63 | 12.28 | 8.02 | 1.73 |
| 20 | 18 | 3000 | 0.2 | 2 | 19.11 | 2338.86 | 0.23 | 2.05 | 6.17 | 22.04 | 15.94 | 2.58 |
| 23 | 18 | 3000 | 0.25 | 2 | 19.25 | 2775.68 | 0.27 | 2.14 | 6.95 | 7.48 | 9.55 | 7.24 |
| 26 | 18 | 3000 | 0.3 | 2 | 19.34 | 2979.92 | 0.30 | 2.43 | 7.43 | 0.67 | 0.53 | 21.44 |
| 29 | 21 | 2000 | 0.2 | 2 | 21.36 | 2485.50 | 0.25 | 2.16 | 1.71 | 24.28 | 25.20 | 8.22 |
| 32 | 21 | 2000 | 0.25 | 2 | 21.04 | 2428.03 | 0.26 | 1.89 | 0.19 | 21.40 | 2.45 | 5.33 |
| 35 | 21 | 2000 | 0.3 | 2 | 20.86 | 2247.92 | 0.23 | 2.01 | 0.68 | 12.40 | 23.43 | 0.65 |
| 38 | 21 | 2500 | 0.2 | 2 | 20.59 | 2063.78 | 0.22 | 1.91 | 1.95 | 17.45 | 8.65 | 4.33 |
| 41 | 21 | 2500 | 0.25 | 2 | 20.42 | 2554.27 | 0.27 | 1.93 | 2.78 | 2.17 | 6.07 | 3.44 |
| 44 | 21 | 2500 | 0.3 | 2 | 19.15 | 2699.99 | 0.28 | 1.81 | 8.83 | 8.00 | 7.79 | 9.51 |
| 47 | 21 | 3000 | 0.2 | 2 | 20.86 | 2321.69 | 0.23 | 2.28 | 0.66 | 22.61 | 13.31 | 14.07 |
| 50 | 21 | 3000 | 0.25 | 2 | 19.61 | 2735.42 | 0.28 | 1.95 | 6.63 | 8.82 | 10.37 | 2.46 |
| 53 | 21 | 3000 | 0.3 | 2 | 19.22 | 2817.49 | 0.28 | 2.17 | 8.45 | 6.08 | 7.73 | 8.46 |
| 56 | 24 | 2000 | 0.2 | 2 | 21.14 | 2383.18 | 0.24 | 2.12 | 11.92 | 19.16 | 20.08 | 6.23 |
| 59 | 24 | 2000 | 0.25 | 2 | 20.50 | 2192.88 | 0.23 | 2.02 | 14.57 | 9.64 | 9.78 | 0.95 |
| 62 | 24 | 2000 | 0.3 | 3 | 21.15 | 2238.57 | 0.22 | 2.20 | 11.88 | 11.93 | 26.49 | 26.78 |
| 65 | 24 | 2500 | 0.25 | 2 | 20.46 | 2150.42 | 0.22 | 1.96 | 14.75 | 13.98 | 10.45 | 1.89 |
| 68 | 24 | 2500 | 0.3 | 3 | 21.15 | 2564.36 | 0.26 | 2.24 | 11.87 | 2.57 | 14.93 | 25.30 |
| 71 | 24 | 3000 | 0.25 | 2 | 20.32 | 2657.45 | 0.28 | 1.74 | 15.33 | 11.42 | 11.59 | 12.77 |
| 74 | 24 | 3000 | 0.3 | 3 | 19.62 | 2745.20 | 0.27 | 2.01 | 18.23 | 8.49 | 8.37 | 32.86 |
|  | | | | | | | | **Average** | 8.75 | 12.24 | 11.65 | 8.89 |

**Supplementary Table 7** Experimental and estimation results using ANFIS (inverse framework)

|  | Experimental results | | | | Estimation results | | | | Percentage errors | | | |
| --- | --- | --- | --- | --- | --- | --- | --- | --- | --- | --- | --- | --- |
| Sample | Laser power (W) | Laser speed (mm/s) | Scan spacing (mm) | Vertical Position | Laser Power | Laser Speed | Scan spacing | Vertical Position | Laser Power (%) | Laser Speed (%) | Scan spacing (%) | Vertical Position (%) |
| 2 | 18 | 2000 | 0.2 | 2 | 17.56 | 2687.00 | 0.20 | 2.06 | 2.46 | 34.35 | 0.295 | 3 |
| 5 | 18 | 2000 | 0.25 | 2 | 19.16 | 2173.00 | 0.25 | 1.88 | 6.43 | 8.65 | 0.2 | 6.2 |
| 8 | 18 | 2000 | 0.3 | 2 | 21.85 | 2985.00 | 0.28 | 2.20 | 21.37 | 49.25 | 5.233 | 10.2 |
| 11 | 18 | 2500 | 0.2 | 2 | 19.64 | 2169.00 | 0.23 | 1.58 | 9.13 | 13.24 | 15.4 | 21 |
| 14 | 18 | 2500 | 0.25 | 2 | 21.19 | 2569.00 | 0.26 | 2.70 | 17.70 | 2.76 | 5.68 | 35 |
| 17 | 18 | 2500 | 0.3 | 2 | 17.81 | 2749.00 | 0.27 | 2.14 | 1.04 | 9.96 | 9.90 | 6.9 |
| 20 | 18 | 3000 | 0.2 | 2 | 18.44 | 2948.00 | 0.25 | 1.51 | 2.44 | 1.73 | 24.75 | 24.6 |
| 23 | 18 | 3000 | 0.25 | 2 | 18.48 | 2737.00 | 0.29 | 0.30 | 2.65 | 8.77 | 17.92 | 84.8 |
| 26 | 18 | 3000 | 0.3 | 2 | 17.20 | 2896.00 | 0.29 | 2.13 | 4.47 | 3.47 | 2.03 | 6.6 |
| 29 | 21 | 2000 | 0.2 | 2 | 21.51 | 2479.00 | 0.26 | 2.58 | 2.43 | 23.95 | 31.25 | 28.8 |
| 32 | 21 | 2000 | 0.25 | 2 | 22.42 | 2488.00 | 0.27 | 1.56 | 6.77 | 24.4 | 8.64 | 21.8 |
| 35 | 21 | 2000 | 0.3 | 2 | 21.27 | 2413.00 | 0.23 | 1.96 | 1.29 | 20.65 | 22.87 | 2.1 |
| 38 | 21 | 2500 | 0.2 | 2 | 25.80 | 2003.59 | 0.25 | 1.97 | 22.86 | 19.856 | 24.55 | 1.4 |
| 41 | 21 | 2500 | 0.25 | 2 | 20.96 | 2562.00 | 0.27 | 1.77 | 0.17 | 2.48 | 6.12 | 11.4 |
| 44 | 21 | 2500 | 0.3 | 2 | 18.56 | 2604.00 | 0.29 | 0.78 | 11.62 | 4.16 | 3.97 | 60.8 |
| 47 | 21 | 3000 | 0.2 | 2 | 19.66 | 2233.00 | 0.24 | 2.67 | 6.40 | 25.57 | 21.2 | 33.6 |
| 50 | 21 | 3000 | 0.25 | 2 | 17.51 | 2725.00 | 0.26 | 2.49 | 16.61 | 9.17 | 2.72 | 24.5 |
| 53 | 21 | 3000 | 0.3 | 2 | 21.58 | 2907.00 | 0.33 | 1.04 | 2.74 | 3.1 | 10.00 | 48.25 |
| 56 | 24 | 2000 | 0.2 | 2 | 21.24 | 2423.00 | 0.25 | 2.32 | 11.5 | 21.15 | 24.7 | 16.1 |
| 59 | 24 | 2000 | 0.25 | 2 | 20.39 | 2282.00 | 0.24 | 1.54 | 15.05 | 14.1 | 5.04 | 22.9 |
| 62 | 24 | 2000 | 0.3 | 3 | 20.36 | 2296.00 | 0.20 | 2.62 | 15.175 | 14.8 | 33.48 | 12.667 |
| 65 | 24 | 2500 | 0.25 | 2 | 20.96 | 2275.00 | 0.24 | 1.40 | 12.65 | 9 | 5.76 | 29.8 |
| 68 | 24 | 2500 | 0.3 | 3 | 20.96 | 2469.00 | 0.27 | 3.06 | 12.65 | 1.24 | 11.33 | 2 |
| 71 | 24 | 3000 | 0.25 | 2 | 22.31 | 2519.00 | 0.27 | 1.85 | 7.025 | 16.03 | 6.28 | 7.4 |
| 74 | 24 | 3000 | 0.3 | 3 | 17.49 | 2839.00 | 0.25 | 2.95 | 27.108 | 5.37 | 16.467 | 1.67 |
|  | | | | | | | | **Average** | 9.59 | 13.89 | 12.63 | 20.94 |
